# Spectral X-Ray CT Image Reconstruction with a Combination of Energy-Integrating and Photon-Counting Detectors

**DOI:** 10.1371/journal.pone.0155374

**Published:** 2016-05-12

**Authors:** Qingsong Yang, Wenxiang Cong, Yan Xi, Ge Wang

**Affiliations:** Department of Biomedical Engineering, Rensselaer Polytechnic Institute, Troy, New York, United States; Zhejiang Univ, CHINA

## Abstract

The purpose of this paper is to develop an algorithm for hybrid spectral computed tomography (CT) which combines energy-integrating and photon-counting detectors. While the energy-integrating scan is global, the photon-counting scan can have a local field of view (FOV). The algorithm synthesizes both spectral data and energy-integrating data. Low rank and sparsity prior is used for spectral CT reconstruction. An initial estimation is obtained from the projection data based on physical principles of x-ray interaction with the matter, which provides a more accurate Taylor expansion than previous work and can guarantee the convergence of the algorithm. Numerical simulation with clinical CT images are performed. The proposed algorithm produces very good spectral features outside the FOV when no K-edge material exists. Exterior reconstruction of K-edge material can be partially achieved.

## Introduction

The x-ray tube spectrum is polychromatic with its energy range typically from 30keV to 140keV. However, with energy-integrating detectors (EIDs) x-ray CT cannot discriminate the energy difference and only reconstructs an image based on a monochromatic x-ray transmission model. The resultant model mismatch leads to the loss of energy-dependent information, such as the K-edge effect, and sometimes severe beam-hardening artifacts in a reconstructed image. On the other hand, photon-counting detectors (PCDs) with the energy-resolving capability are being developed for medical x-ray CT [1]. X-ray CT and micro-CT systems with PCDs have been prototyped for evaluation and refinement [2–6]. There are multiple publications demonstrating the benefits of spectral CT in pre-clinical and clinical application [7–11]. Clearly, spectral CT is a new trend in the CT field to overcome the drawbacks of conventional CT and open a great window of opportunities for improved and new biomedical applications.

However, the performance of PCDs is not impeccable. One of the main limitations of PCDs is that it cannot reach a high counting rate required by current clinical CT. It is reported in [12] and other papers that the counting rate of PCDs is at least an order of magnitude less than that of typical EIDs. While the detection technology is under rapid development, the counting rate remains a critical problem.

For a CT scanner to have an energy-discriminant imaging capability while keeping the system cost within a reasonable range and avoiding the low counting rate, our group built a hybrid micro-CT prototype, which incorporates a narrow PCD array into a traditional EIDs-based micro-CT system [13, 14]. With the EID data, a traditional CT image can be reconstructed. With the PCD data, a spectral field of view (FOV) can be defined, and an interior region of interest (ROI) of an object can be spectrally reconstructed over the spectral FOV using a compressed sensing based statistical interior reconstruction algorithm with the associated traditional CT image as a prior [15]. Interestingly, both simulation and preliminary results have shown that spectral information is rich well beyond the spectral FOV directly covered by the PCDs. Furthermore, in [16] we distributed PCDs along a detector array so that PCDs can more evenly cover the whole object. A split Bregman iterative algorithm was designed to utilize the spectral synergy between the PCD and EID data and the spatial correlation among CT images in different energy bins.

In this paper, we focus on the reconstruction algorithm development assuming the hybrid detector array with a narrow PCD module in the center, which seems the most practical and cost-effective spectral CT architecture option at this stage. In the next section, we compare two hybrid detector array layouts and explain why we choose the first one that has only one PCD module in the center. Then, we describe an image reconstruction algorithm with the sparsity and low-rank prior in which an alternating direction method of multipliers (ADMM) is used for optimization. In the third section, representative numerical results are summarized, with respect to different PCD-to-EID ratios. Also, we analyze the effect of a contrast agent. In the last section, we discuss relevant issues and conclude the paper.

## Materials and Methods

### Hybrid Detector Array Designs

**Fig 1:**
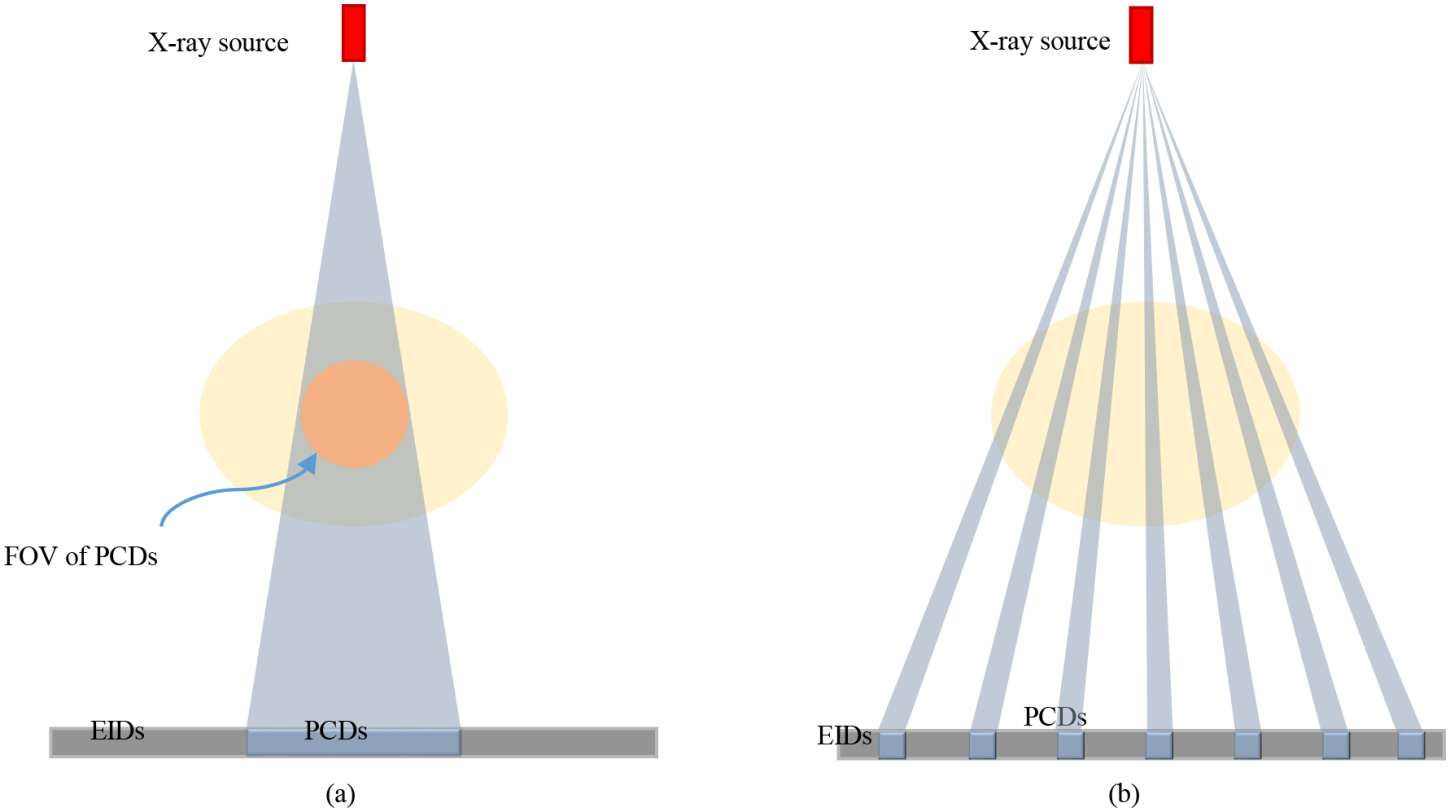
Two proposed hybrid detector array designs. (a) The first design uses a PCD module in the center of the detector panel, and (b) the second design distributes PCD modules across the whole array.

As mentioned earlier, two hybrid detector array designs were considered, the first one restricts the PCDs in the center (Fig 1(a)), and in the second one the PCDs are distributed along the detector array (Fig 1(b)). With the former hybrid design, exact reconstruction can be achieved over the spectral FOV, according to interior reconstruction theory [15]. In the object outside the interior ROI over the spectral FOV defined by the PCDs, the spectral data are acquired in a limited-angle fashion. The further the area is away from the ROI, the less spectral data are acquired. On the other hand, with the latter hybrid design, spectral information is collected over the full FOV defined by the whole detector array. However, if the ratio of PCDs and EIDs is finite, the resolution of a reconstructed spectral image will be compromised relative to that achievable with a full PCD array. Clearly, both hybrid designs face the insufficient data problem when it comes to exact image reconstruction over the full FOV.

As far as the hardware configuration is concerned, placing the PCDs in the center is easy to achieve. Actually, a simple implementation is to use a dual source system as in the prototype system that we built in [13]. In contrast, it is more complicated to mix PCD modules with EID bins since they have different sizes and electric circuits. Moreover, the flux rate mismatch can be handled more easily in the first hybrid design because the attenuation is high through a central ROI compared to the periphery of the ROI, and the dynamic range of the flux rate is limited through the ROI anyway [17]. Taking both the reconstruction and hardware factors into account, we suggest not to use multiple PCD bins with the current PCDs technology, and will focus on the first hybrid detector array design in this paper.

### Image Reconstruction

The projection data acquired in the first hybrid detector scheme are sufficient for theoretically exact interior ROI reconstruction [15] but they are insufficient for exact exterior reconstruction. Hence, EID data are essential to improve image quality of the global image reconstruction. Additional prior knowledge is also required for optimal image reconstruction. In the next two subsections, we explain how the proposed algorithm utilizes PCD and EID data synergistically and how prior constraints are integrated into a unified reconstruction framework.

#### Data Synthesis

The linear attenuation of a monochromatic x-ray beam at the energy *E* is described by the Beer-Lambert law:

$$I\left(E\right)=I_{0}\left(E\right)\text{exp}\left(-\int_{l}\mu \left(x,E\right)dl\right)$$ (1)

where *I*_0_(*E*) is the initial intensity of the x-ray beam before it passes through an object, and *μ*(*x*, *E*) is the linear attenuation coefficient of the object at a position *x*. For EIDs, the intensity over the entire x-ray spectrum is accumulated to give a single readout of the detector:

$$I_{g}=\int_{0}^{E_{max}}I_{0}\left(E\right)Q\left(E\right)\text{exp}\left(-\int_{l}\mu \left(x,E\right)dl\right)dE$$ (2)

where *E*_*max*_ is the maximum energy of the x-ray spectrum, and *Q*(*E*) denotes the energy-dependent detector response. If no object in the x-ray beam, the blank scan of conventional CT is

$$I=\int_{0}^{E_{max}}I_{0}\left(E\right)Q\left(E\right)dE$$ (3)

For PCDs, the spectral range of the x-ray beam can be divided into multiple energy bins, i.e. *E*_*k*_, *k* = 1, 2, …, *M*. In our experiment, we have 8 energy bins from 30keV to 120keV. Each energy window is small enough so that within each energy bin, the energy-dependent attenuation coefficients of the object can be approximated monochromatic and are denoted as *μ*_*j*_(*E*_*k*_), *j* = 1, 2, …, *N*, for each of *N* pixels in an image. Then, the line integral in (Eq 2) is discretized in terms of energy bins by the summation of the image pixels with a weighting factor *a*_*ij*_, where *i* indicates the *i*-th x-ray path and *a*_*ij*_ accounts for the imaging geometry:

$$\int_{l}\mu \left(x,E_{j}\right)dl=\sum_{k=1}^{N}a_{ij}\mu_{j}\left(E_{k}\right)$$ (4)

Thus, we have

$$I_{g}=\sum_{k=1}^{M}I_{0}\left(E_{j}\right)Q\left(E_{j}\right)\text{exp}\left(-\sum_{j=1}^{N}a_{ij}\mu_{j}\left(E_{k}\right)\right)$$ (5)

$$I=\overset{M}{\underset{k=1}{\sum }}I_{0}\left(E_{k}\right)Q\left(E_{k}\right)$$ (6)

Assuming that we have a good estimation of *μ*(*E*_*k*_) (the estimation method will be described below), denoted by $\tilde{\mu }(E_{k})$, we can rewrite (eq 5) as

$$\begin{array}{c} I_{g}=\overset{M}{\underset{k=1}{\sum }}I_{0}\left(E_{j}\right)Q\left(E_{j}\right)\;\text{exp}\left(-\overset{N}{\underset{j=1}{\sum }}a_{ij}\mu_{j}\left(E_{k}\right)+\overset{N}{\underset{j=1}{\sum }}a_{ij}{\tilde{\mu }}_{j}\left(E_{k}\right)-\overset{N}{\underset{j=1}{\sum }}a_{ij}{\tilde{\mu }}_{j}\left(E_{k}\right)\right)\\ =\overset{M}{\underset{k=1}{\sum }}I_{0}\left(E_{j}\right)Q\left(E_{j}\right)\text{exp}\left(-\overset{N}{\underset{j=1}{\sum }}a_{ij}{\tilde{\mu }}_{j}\left(E_{k}\right)\right)\text{exp}\left(\overset{N}{\underset{j=1}{\sum }}a_{ij}{\tilde{\mu }}_{j}\left(E_{k}\right)-\overset{N}{\underset{j=1}{\sum }}a_{ij}\mu_{j}\left(E_{k}\right)\right) \end{array}$$ (7)

If $\tilde{\mu }(E_{k})$ is very close to the true value, their difference is small enough so that we can use the Taylor expansion on the second exponential term. That is, (Eq 7) can be approximated as

$$I_{g}\approx \overset{M}{\underset{k=1}{\sum }}I_{0}\;\left(E_{j}\right)Q\left(E_{j}\right)\text{exp}\left(-\overset{N}{\underset{j=1}{\sum }}a_{ij}{\tilde{\mu }}_{j}\left(E_{k}\right)\right)\left\{1+\overset{N}{\underset{j=1}{\sum }}a_{ij}{\tilde{\mu }}_{j}\left(E_{k}\right)-\overset{N}{\underset{j=1}{\sum }}a_{ij}\mu_{j}\left(E_{k}\right)\right\}$$ (8)

which can be further rewritten as

$$\overset{M}{\underset{k=1}{\sum }}{\tilde{I}}_{i}\left(E_{k}\right)\left[\overset{N}{\underset{j=1}{\sum }}a_{ij}\mu_{j}\left(E_{k}\right)\right]=\overset{M}{\underset{k=1}{\sum }}{\tilde{I}}_{i}\left(E_{k}\right)\left[1+\overset{N}{\underset{j=1}{\sum }}a_{ij}{\tilde{\mu }}_{ij}\left(E_{k}\right)\right]-I_{g}$$ (9)

Where

$${\tilde{I}}_{i}\left(E_{k}\right)=I_{0}\left(E_{j}\right)Q\left(E_{j}\right)\text{exp}\left(-\overset{N}{\underset{j=1}{\sum }}a_{ij}{\tilde{\mu }}_{j}\left(E_{k}\right)\right)$$ (10)

This is the equation for one x-ray beam. When putting all the x-ray beams used in a scan together, (Eq 9) will be the following matrix form:

$$\overset{M}{\underset{k=1}{\sum }}I_{k}A_{g}\mu_{k}=Y_{g}$$ (11)

where ***A***_***g***_ is the system matrix for grayscale scan, the left side of the above equation is the weighted summation of the forward projection, and the right side ***Y***_***g***_ is the modified photon intensity as defined by the right side of (Eq 9).

For the *j-th* channel of PCDs, (Eq 7)is easily obtained as follows:

$$I_{j}=I_{0}\left(E_{j}\right)Q\left(E_{j}\right)\text{exp}\left(-\overset{N}{\underset{i=1}{\sum }}a_{i}\mu_{i}\left(E_{j}\right)\right)$$ (12)

thus,

$$\overset{N}{\underset{i=1}{\sum }}a_{i}\mu_{i}\left(E_{j}\right)=-\text{ln}\frac{I_{j}}{I_{0}\left(E_{j}\right)Q\left(E_{j}\right)}$$ (13)

This can be rewritten into the following matrix equation:

$$A_{c}\mu_{k}=Y_{c,k}$$ (14)

where ***A***_***c***_ accounts for the system geometry of photon-counting detectors for color/spectral information. Based on Eqs (11) and (14), we establish the objective function for our hybrid CT system in the following general form:

$$\underset{\mu_{k}}{\text{min}}\left[\overset{M}{\underset{k=1}{\sum }}∥A_{c}\mu_{k}-Y_{c,k}{∥}_{\;2}^{2}+∥\overset{M}{\underset{k=1}{\sum }}I_{k}A_{g}\mu_{k}-Y_{g}{∥}_{\;2}^{2}+Prior\left(\mu_{k}\right)\right]$$ (15)

where *Prior*(·) represents the regularization term based on the prior information of the attenuation images.

#### Hybrid Reconstruction with PRISM

Because the PCDs are placed in the central portion of the detector array, its FOV only covers a central region of an object. Hence, exterior reconstruction outside the spectral FOV is difficult and require strong prior knowledge. Thanks to the correlation among the attenuation coefficients in spatial neighborhoods and across energy bins, we can use the prior rank, intensity and sparsity model (PRISM) as the regularization term [18].

Let ***X*** = **[*μ***_**1**_, ***μ***_**2,**_ **…, *μ***_***M***_**]** and ***Y***_***c***_ = [***Y***_**c1**_, ***Y***_**c2**_, **…**, ***Y***_***cM***_]. The PRISM model assumes that ***X*** is the sum of a low rank matrix ***X***_***L***_ and a sparse matrix ***X***_***S***_:

$$X=X_{L}+X_{S}$$ (16)

Then, our optimization problem can be rewritten as

$$\begin{array}{c} \underset{X_{L},X_{S},X}{min}∥A_{c}X-Y_{c}{∥}_{\;F}^{2}+∥A_{g}X-Y_{g}{∥}_{\;F}^{2}+\lambda_{1}∥X_{L}{∥}_{\;*}+\lambda_{2}∥X_{S}{∥}_{\;1}\\ s.t.\;\;X_{L}+X_{S}-X=0 \end{array}$$ (17)

where $A_{g}$ is a linear operator for the second term in (Eq 15) and ‖·‖_*F*_ is the Frobenius norm. To solve the above problem, we utilize an extended ADMM method [19–22] for its simple and easy implementation.

Specifically, we first introduce an auxiliary variable ***D*** and update ***X*** by solving the augmented Lagrangian

$$\underset{X}{\text{min}}\left[∥A_{c}X-Y_{c}{∥}_{\;F}^{2}+∥A_{g}X-Y_{g}{∥}_{\;F}^{2}+\lambda ∥X_{L}+X_{S}-X+D{∥}_{\;F}^{2}\right]$$ (18)

Then, we update ***X***_***L***_ and ***X***_***S***_ by solving

$$X_{L}=\;\underset{X_{L}}{\text{min}}\left[\lambda_{1}∥X_{L}{∥}_{*}+\lambda ∥X_{L}+X_{S}-X+D{∥}_{\;F}^{2}\right]$$ (19)

$$X_{S}=\underset{X_{S}}{\text{min}}\left[\lambda_{2}∥X_{S}{∥}_{1}+\lambda ∥X_{L}+X_{S}-X+D{∥}_{\;F}^{2}\right]$$ (20)

separately. The nuclear norm minimization can be solved using a singular value threshold (SVT) method [23]:

$$X_{L}:=SVT\left(-X_{S}+X-D,\frac{\lambda_{1}}{2\lambda }\right)$$ (21)

while the *l*_1_-norm minimization can be solved using a similar soft-threshold method [24]

$$X_{S}:=Soft\left(-X_{L}+X-D,\frac{\lambda_{2}}{2\lambda }\right)$$ (22)

Finally, we update ***D*** as

$$D:=D+X_{L}+X_{S}-X$$

Since we have used a Taylor approximation in (Eq 8), we should keep updating this approximation during the iterative process. In Table 1, we summarize our iterative method for clarity.

**Table 1:** PRISIM reconstruction with iteratively updated taylor approximation.

Algorithm 1. Hybrid PRISM reconstruction
1. Set λ, λ_1_, and λ_2_
2. Initialize ***X = X***^***initial***^, ***X***_***L***_ **= *X*, *X***_***S***_ **= 0**
3. For i = 1,2,3, …, maximum number of iteration do
a. Update ***Y***_***g***_ according to (Eq 9)
b. Set ***D = X***_***L***_ ***+ X***_***S***_ **− *X***
c. For j = 1,2,3,…,5 do
i. Reconstruct an image by (Eq 18)
ii. Update $X_{L}\;≔\;SVT({-X}_{S}+X-D,\frac{\lambda_{1}}{2\lambda })$
iii. Update $X_{S}\;≔\;Soft({-X}_{L}+X-D,\frac{\lambda_{2}}{2\lambda })$
iv. Update ***D* ≔ *D + X***_***L***_ ***+ X***_***S***_ **− *X***
4. End loop upon sufficiently high data fitting and/or maximum number of iterations

In all of our experiments, we chose *λ*_1_ = *λ*_2_ = 0.01 and *λ* = 1 and terminated the iteration after 1000 times.

#### Initial estimation

The Taylor expansion in (Eq 7) requires a close approximation of the true attenuation image, which can be obtained from the available projection data. As we know, photoelectric and Compton scattering effects are the dominant contrast mechanisms in the diagnostic x-ray energy range. Hence, the x-ray attenuation is decomposed into photoelectric absorption and Compton scattering components:

$$\mu \left(r,E\right)=\frac{\rho N_{A}}{A}\left(\sigma_{ph}+\sigma_{co}\right)$$ (23)

where *ρ*, *N*_*A*_, and *A* are the mass density, Avogadro’s number, and atomic mass, respectively. *σ*_*ph*_ is the photoelectric absorption cross section, and is formulated as $Z^{4}\alpha^{4}\frac{8}{3}\pi r_{\epsilon }^{2}\sqrt{\frac{32}{\epsilon^{7}}}\;$ for *ϵ* < 1 [25], and *Z* is the atomic number, *α* is the fine-structure constant (≈ 1/137), and *r*_*ϵ*_ is the classical radius of an electron (*r*_*ϵ*_ = 2.818*fm*). *σ*_*co*_ is the Compton scattering cross section and can be modeled as *Zf*_*kn*_, where *f*_*kn*_ is the Klein-Nishina function [26]

$$f_{kn}\left(\epsilon \right)=2\pi r_{\epsilon }^{2}\left\{\frac{1+\epsilon }{\epsilon^{2}}\left[\frac{2\left(1+\epsilon \right)}{1+2\epsilon }-\frac{1}{\epsilon }\text{ln}\left(1+2\epsilon \right)\right]+\frac{1}{2\epsilon }\text{ln}\left(1+2\epsilon \right)-\frac{1+3\epsilon }{{\left(1+2\epsilon \right)}^{2}}\right\}$$ (24)

where *ϵ* = *E*/511*keV* gives the energy dependence of the Compton scattering. In this way, (Eq 23) is reduced to

$$\left(r,E\right)=\mu_{\text{eff}}\left(r\right)\left(N_{A}\alpha^{4}\frac{8}{3}\pi r_{\epsilon }^{2}\sqrt{32}\frac{Z_{eff}^{3}}{\epsilon^{3.5}}+N_{A}f_{kn}\left(\epsilon \right)\right)$$ (25)

wher*e $\mu_{eff}\;=\;\frac{\rho Z}{A}$* is an effective electron density, which is independent on energy, and describes structural variations. Mass density is primarily about the morphology of the absorber. The atomic number reflects the number of electrons per atom. *Z*_*ef f*_ is approximated by the atomic number of water in the initial estimation of the linear attenuation coefficients of the object. Inserting (eq 25) into (eq 2), we have,

$$I=\overset{E_{max}}{\underset{0}{\int }}I_{0}\left(E\right)Q\left(E\right)\text{exp}\left(-\left[N_{A}\alpha^{4}\frac{8}{3}\pi r_{\epsilon }^{2}\sqrt{32}\frac{Z_{eff}^{3}}{\epsilon^{3.5}}+N_{A}f_{kn}\left(\epsilon \right)\right]\int_{l}\mu_{ef\;f}\left(r\right)dr\right)dE$$ (26)

The right hand side of the above equation is a monotonous decreasing function with respect to the variable ∫_*l*_ *μ*_eff_(*r*)*dr*, and (Eq 26) has a unique solution that can be determined using an optimization method from measured *I* and *I*_0_. Therefore, *μ*_eff_(*r*) can be reconstructed, and an image at any specific energy can be estimated via (Eq 25).

### Experimental Design

A 2D fan-beam geometry was assumed, in which 180 projection angles were uniformly specified from 0° to 360°. The x-ray source was placed 50cm away from the iso-center of the imaging plane, while the detector array was 50cm away from the center on the opposite side. The detector array consisted of 512 elements, which were equi-spatially distributed, covering 70cm in total length. We define a photon-counting to energy-integrating extent ratio (PEER) in terms of detector elements array length:

$$PEER=\frac{PCD\;array\;length}{EID\;array\;length}$$

In the experiments, we tested different cases and will only report our representative results for $PEER\;=\;1,\frac{1}{2},\frac{1}{3}$, respectively.

**Fig 2:**
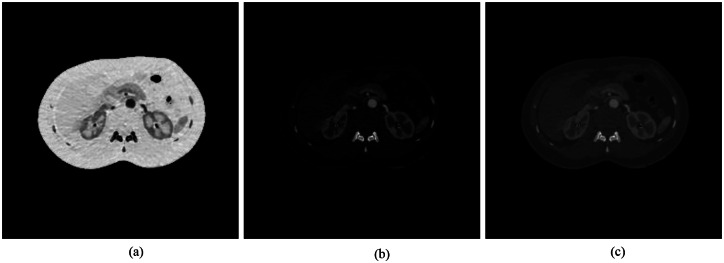
Realistic numerical phantom from clinical CT datasets. (a) Water and (b) bone material basis functions obtained from a GE Discovery CT750 scanner, and (c) the mono-energy image in the fourth energy channel. The display windows are [0, 4.16], [0, *10*], and [0, 0.23] for water, bone and the mono image, respectively.

**Fig 3:**
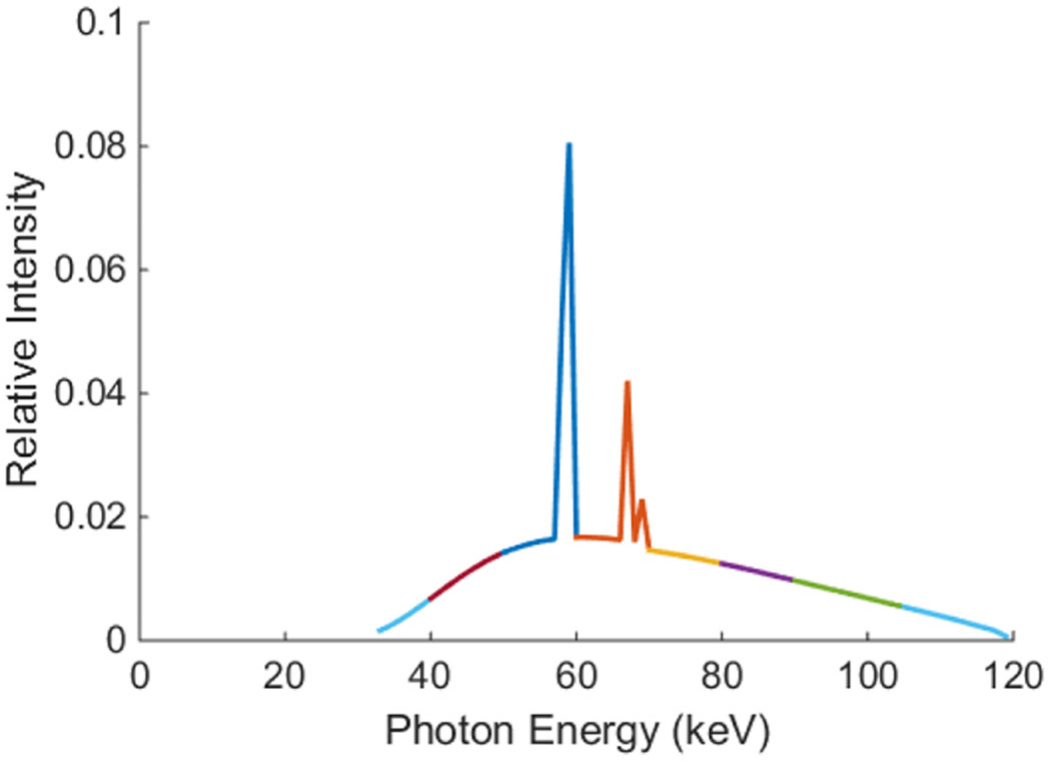
X-ray spectrum generated using an open source software package [27]. The different colors indicate various energy bins/channels.

Dual-energy CT datasets of the human abdomen provided by Shanghai Ruijin Hospital, which were collected on a GE Discovery CT750 scanner were used to construct our realistic numerical phantom. (Identifying patient information was removed from the CT images. We consulted Shanghai Ruijin Hospital and obtained approval for the retrospective use of these data in a research study.) Then, two material bases, bone and water, were reconstructed from the DECT datasets. Fig 2(a) and 2(b) shows the two material bases. An x-ray spectrum was generated using an open source software package [27]. The broad x-ray spectrum was divided into 8 narrow energy bins/channels, as shown in Fig 3. For each energy bin, we constructed a narrow-energy image using the two material bases. Fig 2(c) displays a typical mono-energy image in the fourth energy channel.

## Results and Discussion

**Fig 4:**
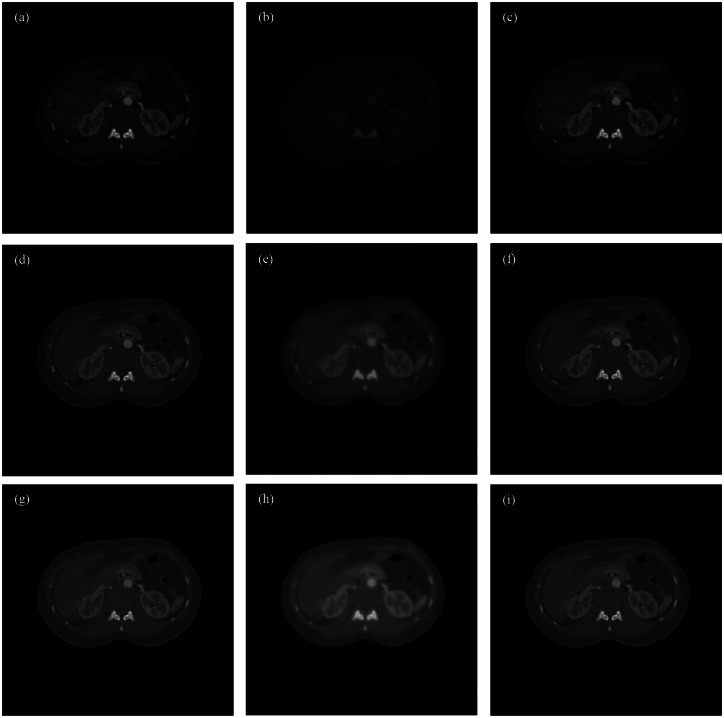
Comparison of the reconstructed images in the case of *PEER* = 1. From top to bottom are the images in the 1^st^, 4^th^, and 8^th^ energy channels respectively. From left to right are the ground truth, initial estimation, and final reconstruction respectively. Note that the middle estimation images are in the same display window as the ground truth and reconstructed results. The estimation was initially not accurate for low-energy images. The display windows are [0, 0.56], [0, *23*], and [0, 0.16] in linear attenuation coefficient for the 1^st^, 4^th^, and 8^th^ energy channels respectively.

**Fig 5:**
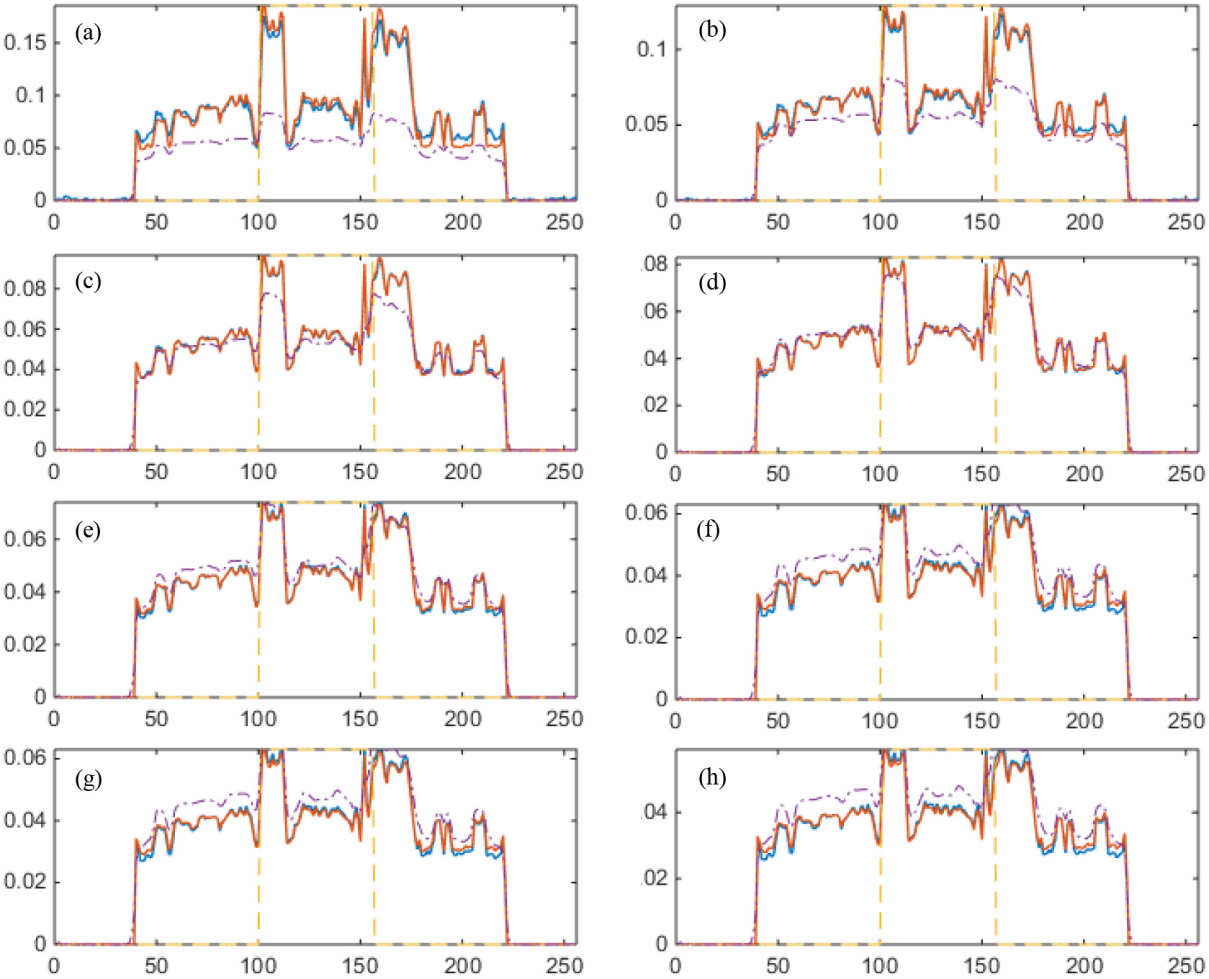
Comparison of the reconstructed profiles versus the ground truth along the central horizontal line when $PEER\;=\;\frac{1}{3}$ without data noise. The blue solid lines are the profiles through the reconstructed images. The purple dash-dot ones are the initial estimation. The red solid ones are the ground truth. The yellow dash ones indicate the FOV defined by the PCDs. From (a) to (h) are the results in the 1^st^ to 8^th^ energy channels respectively.

**Fig 6:**
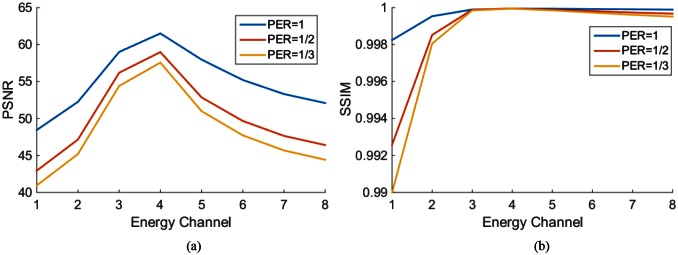
PSNR and SSIM plots in each energy channel when $PER\;=\;1,\frac{1}{2},\frac{1}{3}$ respectively without data noise.

**Fig 7:**
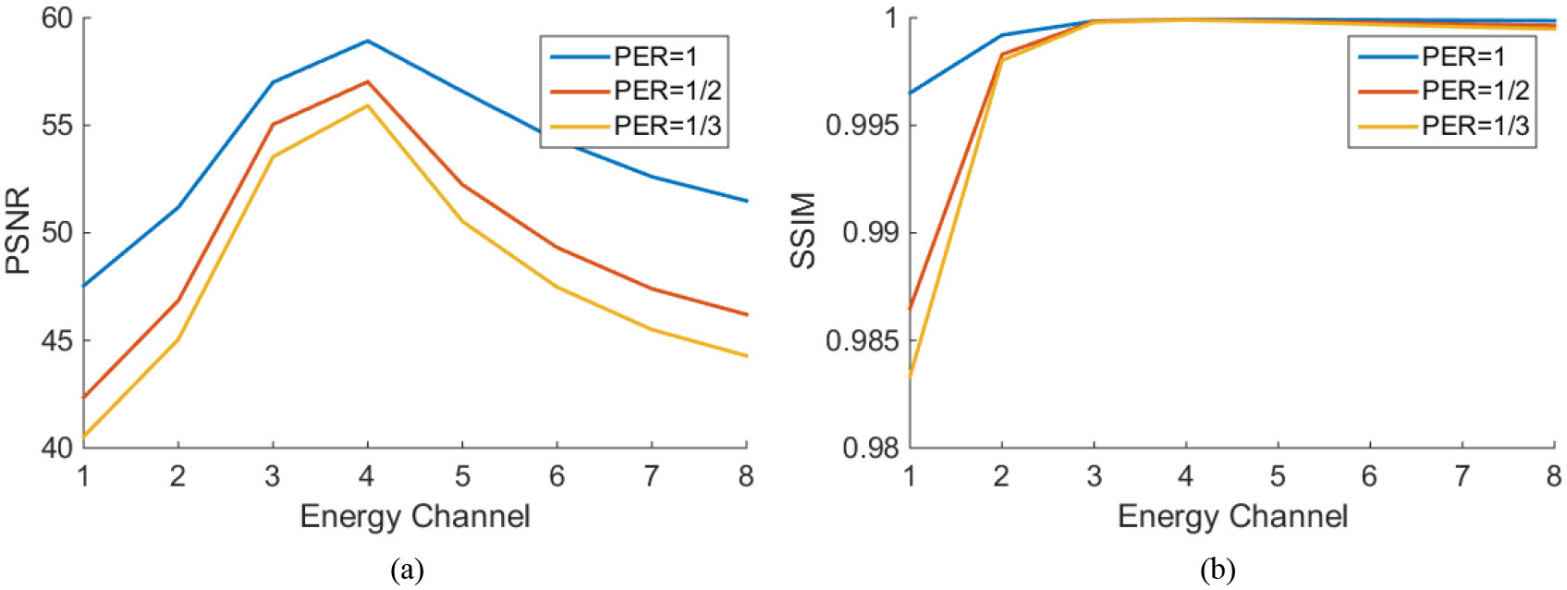
PSNR and SSIM plots in each energy channel when $PER\;=\;1,\frac{1}{2},\frac{1}{3}$ respectively with Poisson noise added to the ideal projection data.

We first performed a noise-free experiment to validate the proposed algorithm. Fig 4 shows the ground truth, the initial estimated image, the reconstructed mono-energy images in the 1^st^, 4^th^, and 8^th^ energy channels respectively for the PEER ratio being 1. In Fig 5, the profiles are along the central horizontal line through these mono-energy images when the PEER ratio was 1/3. To quantify the reconstruction results, we calculated the peak signal-to-noise ratio (PSNR) and the structural similarity index measure (SSIM) [28] between the reconstructed image and the phantom across the entire image region for each energy channel. Fig 6(a) plots the PSNR versus the energy channels for $PER\;=\;1,\frac{1}{2},\frac{1}{3}$. Fig 6(b) shows the corresponding SSIM plots. Next, we added Poisson noise to the data assuming the incident photon *I* = 1 × 10^7^ and reeated the image reconstructions. Fig 7 gives the resultant PSNR and SSIM plots.

**Fig 8:**
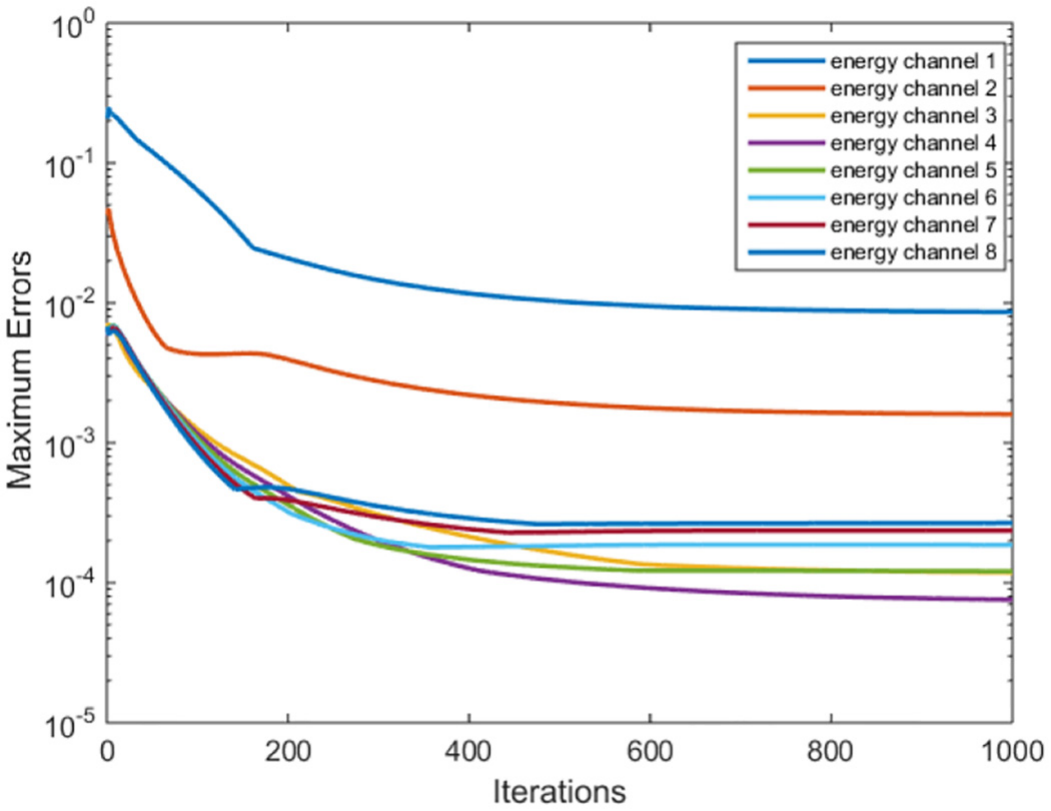
Convergence analysis. Maximum quadratic errors $\max_{i}{\left[\sum_{j\;=\;1}^{N}a_{ij}{\tilde{\mu }}_{j}(E_{k})-\sum_{j\;=\;1}^{N}a_{ij}\mu_{j}(E_{k})\right]}^{2}$ in each energy channel versus the number of iterations in the case of *PEER* = 1.The y-axis is on a log scale.

The first-order Taylor expansion in (Eq 8) requires that the errors $\sum_{j\;=\;1}^{N}a_{ij}{\tilde{\mu }}_{j}(E_{k})-\sum_{j\;=\;1}^{N}a_{ij}\mu_{j}(E_{k})$ must be sufficiently small. Taking the experiment with *PEER =* 1 as an example, we calculated the quadratic term ${\left[\sum_{j\;=\;1}^{N}a_{ij}{\tilde{\mu }}_{j}(E_{k})-\sum_{j\;=\;1}^{N}a_{ij}\mu_{j}(E_{k})\right]}^{2}$ in the iterative process. Fig 8 plots the maximum quadratic error in each energy channel versus the number of iterations, which clearly indicates the convergence of the algorithm.

**Fig 9:**
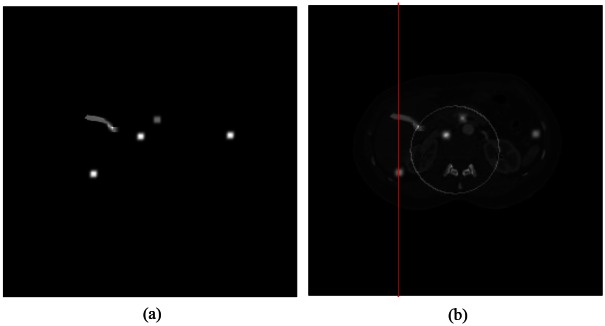
Contrast enhanced CT study. (a) Gadolinium in four spots and one strip (display window [0, 0.04]), and (b) the mono-energy image in the fourth energy channel with additional Gadolinium features (display window [0, 0.23] in linear attenuation coefficient) and the white circle indicates the spectral FOV in the case of $PEER\;=\;\frac{1}{2}$.

To show the benefit of spectral CT relative to DECT, an extra contrast agent basis function was added to the phantom (shown in Fig 9(a)). The new material was introduced into the image as four small spots and one strip of Gadolinium whose K-edge is 50.207keV. Fig 9(b) shows a mono-energy image with the additional Gadolinium basis.

**Fig 10:**
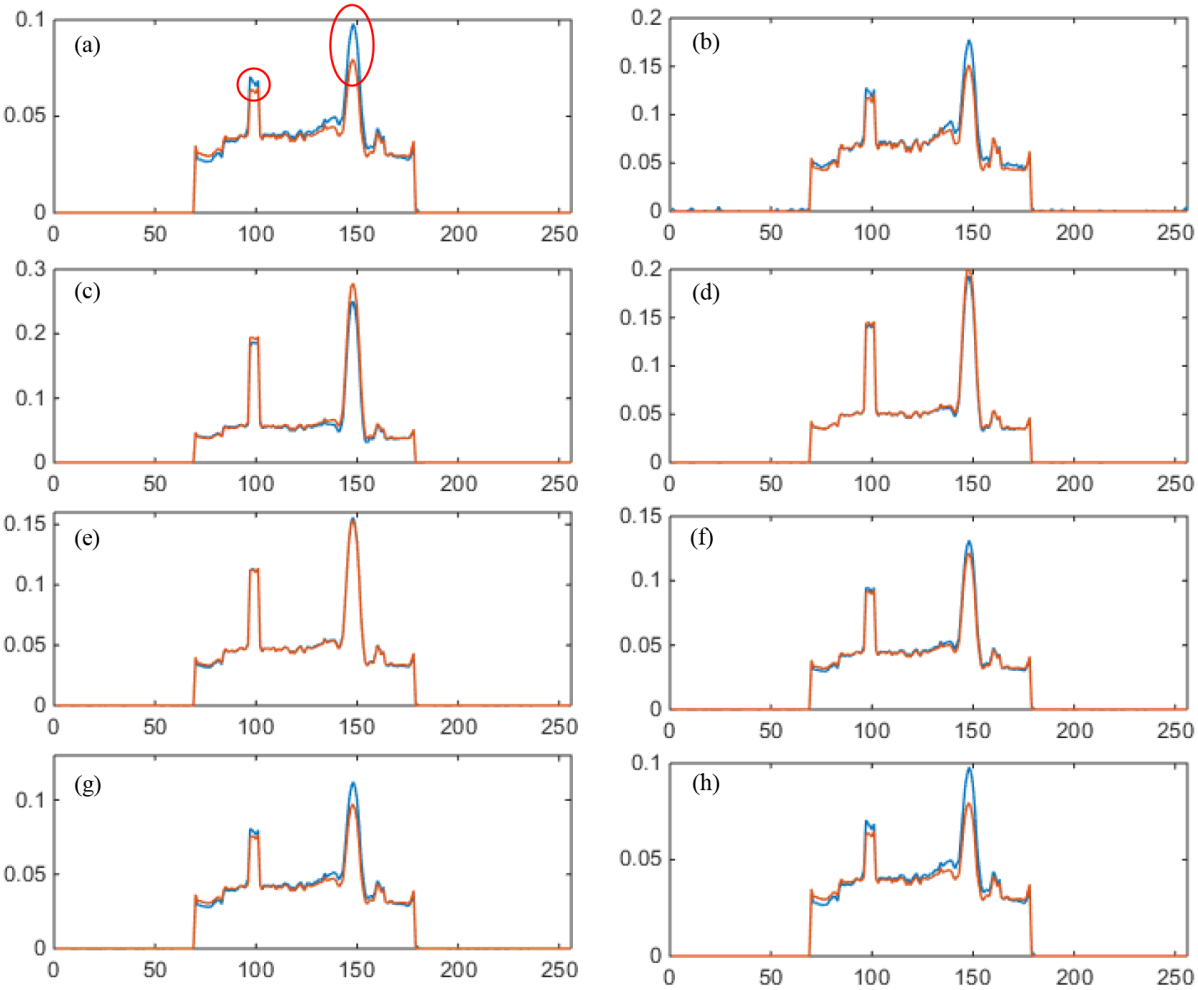
Comparison of the reconstructed profiles versus the ground truth when $PEER\;=\;\frac{1}{2}$ without data noise. The blue solid lines are the reconstructed profiles; while the red solid ones are the ground truth. All the pixels are outside the spectral FOV/ROI defined by the PCDs. From (a) to (h) are the results in the 1^st^ to 8^th^ energy channels respectively. The two red ellipses in (a) show the Gadolinium clusters.

A noiseless experiment with $PEER\;=\;\frac{1}{2}$ was performed. The spectral FOV/ROI defined by the PCDs is in the white circle in Fig 9(b). In Fig 10, the profiles along the vertical red line in Fig 9(b) are totally outside the spectral FOV.

**Fig 11:**
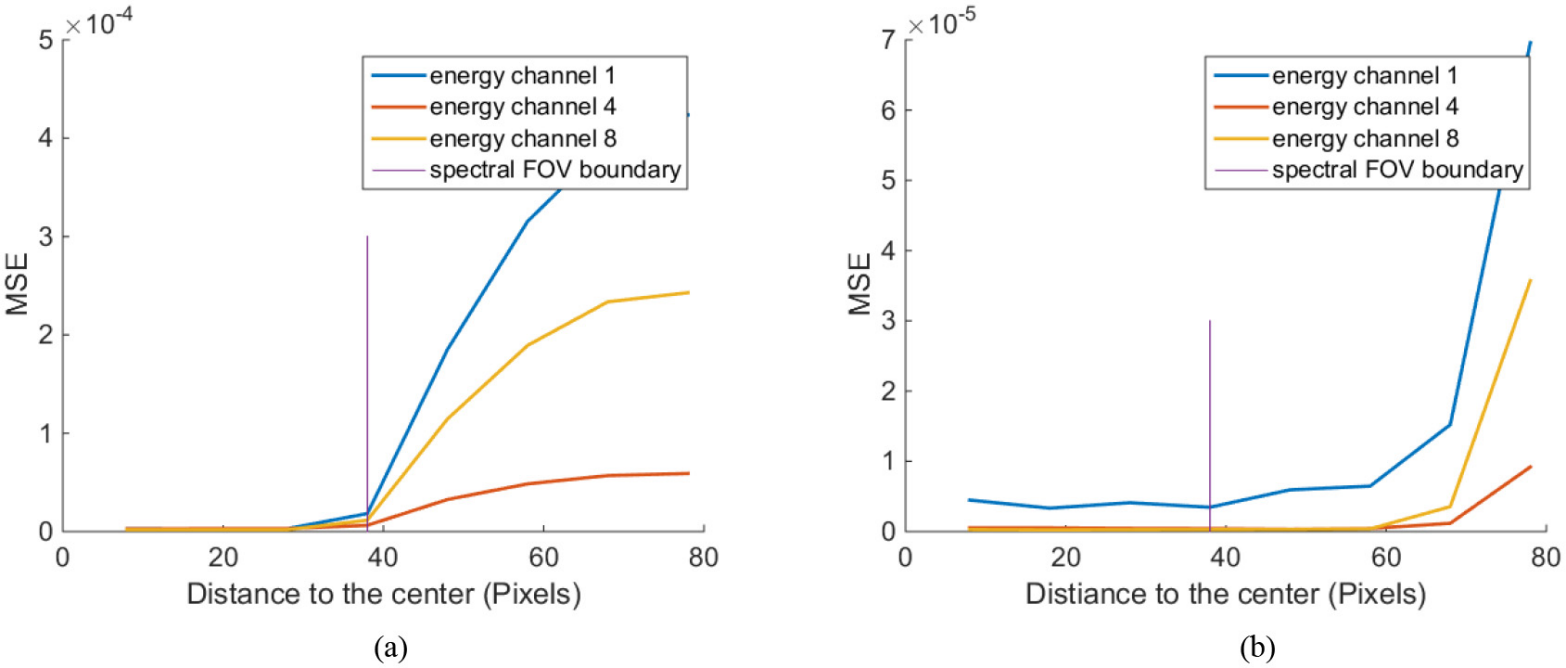
Gadolinium reconstruction errors versus its distance to the center in the case of $PEER\;=\;\frac{1}{2}$. (a) The results in the case of the Gadolinium spot, and (b) is the results in the case of a Gadolinium strip.

To further evaluate the reconstruction errors of the Gadolinium spots and strip, we performed more experiments in which Gadolinium features were placed in different positions. First, we put one Gadolinium spot at the center of the phantom and gradually moved it to the left side along the horizontal line. The experiments with $PEER\;=\;\frac{1}{2}$ were performed for each position of the Gadolinium spot. For all the reconstruction results, we calculated the mean squared errors (MSE) of the Gadolinium spots. Fig 11(a) plots the reconstruction error of the Gadolinium spot versus the position in the phantom. Similarly, a horizontal strip crossing the spectral FOV was added to the original phantom and we repeated the just-described experiment. Fig 11(b) shows the reconstruction error versus the position of the Gadolinium strip.

The simulation results demonstrate an overall promising performance of our proposed algorithm for hybrid spectral CT. The profiles in Figs 5 and 10 show that the pixel values match well between the truth and reconstruction. Although our initial estimation was not perfect, the errors were effectively corrected by the proposed algorithm. Also, the quantitative measurements in terms of PSNR (greater than 45) and SSIM (not less than 0.98) indicate an excellent image quality insensitive to data noise.

In (Eq 8), we have taken the first-order Taylor expansion when $\sum_{j\;=\;1}^{N}a_{ij}{\tilde{\mu }}_{j}(E_{k})-\sum_{j\;=\;1}^{N}a_{ij}\mu_{j}(E_{k})$ is small. Mathematically, we have not arrived a general answer yet as to how small the value must be. As seen in Fig 8, the initial estimation gave a sizable error. However, as the iteration process continues, the error gets reduced to be rather small (below 10^−3^ in most energy bins). It is desirable that a good initial estimation is used to improve the reconstruction quality and accelerate the convergence speed.

In Fig 5, the spectral FOV is only about one third of the whole FOV, but it is still seen that the parts outside the spectral FOV can be well reconstructed. Fig 10 demonstrates such an exterior spectral reconstruction from limited angle spectral data and global grey-scale information. According to these profiles, when the pixels are further away from the spectral FOV, the reconstructed pixel values are subject to larger errors. This is expected since spectral data for these pixels are collected from a more seriously limited angular range. Nevertheless, the results are acceptable as long as the relative errors are within reasonable range, which is the case in a substantially sized neighborhood around the spectral FOV.

In Figs 6 and 7, the image quality in different energy channels are not the same. The results in the 3^rd^, 4^th^, and 5^th^ energy channels outperform that in the other channels. The non-uniformity is more noticeable in Fig 10 when we compare the two red circles in different energy bins. A reason behind this phenomenon is the errors in the initial estimation. As shown in Fig 8, the initial errors are much greater for the 1^st^ and 2^nd^ energy bins than those for other energy bins. The proposed algorithm utilizes the PCD data for interior spectral image reconstruction and the EID and PCD data for exterior reconstruction according to Eqs (9) (10) and (11). In these equations, the photon numbers in various channels, ${\tilde{I}}_{i}(E_{k})$, play a role of weighting factors. From the x-ray spectrum, it is clear that most of the photons concentrate in the 3^rd^, 4^th^, and 5^th^ energy channels. Because the attenuation coefficients are larger for lower x-ray energy than higher energy, ${\tilde{I}}_{i}(E_{k})$ is rather small in the 1^st^ and 2^nd^ energy channels. Yet, we believe that the overall image quality and the three out of eight energy channels ought to be enough for most imaging tasks.

The reconstruction of Gadolinium is not as compromised outside the spectral FOV. The red peaks in Fig 10(a) indicate where Gadolinium concentrates. Errors around the right peak are substantial, despite that the reconstructed values around the left peak are within a reasonable range. The difference between these two peaks lies in that the left one belongs to a Gadolinium strip whose tail is inside the spectral FOV, while the right one is isolated from the spectral FOV. Furthermore, the reconstruction error plots in Fig 11 also indicate a better reconstruction can be obtained when a continuous strip overlaps the spectral FOV than that associated with an isolated spot. All these results confirms the concept of spectral information diffusion which was mentioned in [14].

## Conclusion

In this study, we have made a significantly improvement on hybrid spectral CT based on the EID and PCD technology relative to our previous publications [13, 14, 16]. A linear relationship has been formulated between gray-scale intensity measurements and spectral components of an underlying image using the Taylor approximation of the polychromatic Beer-Lambert formula. Based on the system of coupled linear equations from gray-scale and spectral measurements, a CS-based iterative algorithm has been proposed to optimize image reconstruction. The initial image is estimated based on the physical principles of x-ray interaction with matter, outperforming the previous work by facilitating the convergence of the algorithm. We have analyzed and compared the two hybrid detector array schemes: the first one puts PCDs in the center while the second one mixes PCDs and EIDs. We have recommended the first design. The numerical tests have shown that the proposed iterative algorithm has an excellent performance, with excellent spectral image information within the spectral FOV and beyond. When there is no K-edge material, the reconstructed images have PSNR greater than 45 and SSIM not less than 0.98. When K-edge material exists outside the spectral FOV, the reconstruction quality is compromised outside the spectral FOV which is still within a reasonable range in most of the energy bins. Finally, further improvements are certainly possible for even better image initiation and iterative reconstruction. We are working along this direction towards practical applications.
